# Plasma Synuclein Aggregate Detection via Constant Shake‐Induced Conversion: A Novel Biomarker for Parkinson's Disease Diagnosis and Progression

**DOI:** 10.1002/alz70856_106147

**Published:** 2026-01-08

**Authors:** Nayoung Ryoo, Hyo Rim Ko

**Affiliations:** ^1^ Eun‐pyeong St. Mary's Hospital of the Catholic University of Korea, Seoul, Seoul, Korea, Republic of (South); ^2^ PeopleBio, Sungnam‐si, Gyeonggi‐do, Korea, Republic of (South)

## Abstract

**Background:**

Synuclein aggregates are a hallmark of synucleinopathies, including Parkinson's disease (PD), dementia with Lewy bodies (DLB), and multiple system atrophy (MSA), contributing to their pathogenesis. Current detection methods rely on cerebrospinal fluid and tissue samples, limiting clinical utility. A robust plasma‐based assay is needed for non‐invasive biomarker development. This study validates the constant shake‐induced conversion (CSIC) method for quantifying plasma synuclein aggregates and evaluates its ability to distinguish PD patients from healthy controls (HCs), demonstrating broader applicability across synucleinopathies.

**Methods:**

The CSIC method was assessed using synuclein seeds incubated for six days. Plasma samples from PD and HC patients were analyzed, and the CSIC method was verified based on synuclein depletion, quantification with the enzyme‐linked immunosorbent assay, and confirmation using Western blotting. Diagnostic accuracy was evaluated using receiver operating characteristic (ROC) analysis, while Spearman correlation coefficients (r) were used to evaluate the association between clinical features and synuclein aggregates. Repeatability and reproducibility were tested by three experimenters.

**Results:**

CSIC effectively amplified synuclein aggregates in plasma, with a significant difference observed between the PD and HC groups (*p* < 0.0001). Synuclein depletion confirmed the specificity of CSIC, while ROC analysis produced an area under the curve of 0.91, with a sensitivity of 80.95% and a specificity of 85% when distinguishing PD patients from HCs. The proposed method also had a coefficient of variation (CV) of less than 10% for repeatability and a CV of around 20% for reproducibility over 48 h. CSIC had a strong clinical correlation with the Hoehn and Yahr stage (*r* = 0.69), the Unified Parkinson's Disease Rating Scale (*r* = 0.68), and the Montreal Cognitive Assessment score (*r* = –0.47).

**Conclusion:**

This study is the first to demonstrate the detection of plasma synuclein aggregates using CSIC, addressing a critical diagnostic gap in synucleinopathies. The ability of CSIC to differentiate PD patients from HCs highlights its potential as a non‐invasive diagnostic tool that advances the use of plasma synuclein aggregates in the diagnostic and therapeutic monitoring of PD and related disorders.

